# The lasting smell of emotions: The effects of reutilizing fear sweat samples

**DOI:** 10.3758/s13428-020-01412-5

**Published:** 2020-05-21

**Authors:** Nuno Gomes, Fábio Silva, Gün R. Semin

**Affiliations:** 1 William James Center for Research, ISPA – Instituto Universitário, Rua Jardim do Tabaco 34, 1149-041 Lisbon, Portugal; 2 William James Center for Research, Department of Education and Psychology, University of Aveiro, Aveiro, Portugal; 3 Faculty of Social and Behavioral Sciences, Utrecht University, Utrecht, the Netherlands

**Keywords:** Human olfaction, Body odors, Fear, Fear sweat, Reuse, Facial EMG

## Abstract

A growing body of research has shown that human apocrine sweat carries information about the emotional state of its donor. Exposure to sweat produced in a fear-inducing context triggers in its receivers a simulacrum of this emotional state, as evidenced by increased *medial frontalis* and *corrugator supercilii* (facial electromyography; fEMG) activity – two facial muscles involved in the display of fear facial expressions. However, despite the increased interest in the effects of emotional sweat, little is known about the properties of these chemical sweat samples. The goal of this study was to examine whether a second application of the same sweat sample would yield reliable results. Specifically, we assessed whether sweat samples collected from Portuguese males (*N =* 8) in fear (vs. neutral)-inducing contexts would produce similar fEMG activations (i.e., in the *medial frontalis* and *corrugator supercilii*) in female receivers (*N =* 60) across two independent applications (the first with Dutch and the second with Portuguese receivers). Our findings showed that exposure to fear (vs. neutral) sweat resulted in higher activation of both muscles compared with neutral odors, revealing a similar data pattern across the two applications and underlining the feasibility of reusing emotional sweat samples. The implications of these findings for properties of these sweat volatiles are discussed.

## Introduction

Compared with other senses such as vision or hearing, human olfaction has largely been neglected. This started to change in the late 1970s (e.g., Russell, 1976), and interest among the scientific community in the human sense of smell has continued to grow since then. One of the research fields in this area that has experienced more development over the past few years is the study of the social communicative function of human body odors (i.e., chemosignals; Semin & de Groot, 2013). However, while human chemosignals, as the medium carrying a wealth of information, is receiving increasing attention, the carrier itself, sweat, is relatively neglected. An important question with considerable practical and theoretical relevance is how long sweat retains its message-carrying function. In other words, how many times can the same sweat sample be used? To answer this question, we first provide a brief overview of the communicative function of human chemosignals leading to the main focus of our research: how durable are the message-carrying properties of emotional body odor samples?

The accumulating research on the effects of human chemosignals on recipients has revealed that these volatiles carry a wide range of information. For instance, human chemosignals have been demonstrated to convey information about age (Mitro et al., 2012), gender (Penn et al., 2007), health status (Olsson et al., 2014), familiarity (e.g., Lundström et al., 2008), reproductive state (Stern & McClintock, 1998), genetic relatedness (Porter, 1998), and affective states (e.g., Chen et al., 2006). Indeed, in the case of affective states, recent studies have shown that chemosignals induced during emotional states lead to a simulacrum of the donor’s emotional state (e.g., fear and happiness; de Groot et al., 2015). Moreover, they modulate a wide range of behavioral responses including mimicry of the donor’s facial expression (see de Groot et al., 2017). For instance, exposure to fear chemosignals results in the activation of the *medial frontalis* and *corrugator supercilii* (de Groot et al., 2014), facial muscles associated with the expression of fear (see Fridlund & Cacioppo, 1986).

While the study of the communicative function of human odors produced while experiencing emotional states has grown considerably, there remain many challenges about how to handle sweat samples (e.g., Parma et al., 2017). One of these is addressed by controlling the bodily conditions to reduce variability between donors as much as possible during the collection of sweat samples. This involves restrictions on, for instance, daily habits of donors (dietary, hygienic, and social restrictions; e.g., Havlíček & Lenochova, 2006). The medium by which the odors are collected is another item (e.g., t-shirts or pads; Roberts et al., 2005). Similarly, the length of the sampling process (i.e., duration of the collection; Havlíček et al., 2006) and how the sample is stored (e.g., the time that samples spend in a freezer; see Lenochova et al., 2009) are issues that have been addressed. These are all crucial factors that can affect the final odor sample, and implementing all these constraints is an expensive process, in terms of both the time and monetary costs involved.

Common practice is to use a sweat sample only once. In other words, once a sweat sample has been used for a specific participant, that sample is normally not used again. The assumption driving this is that the properties of the volatiles responsible for whatever effect is being examined might be reduced or have dissipated. Few studies have examined this question directly. In a paper assessing the effects of freezing plain sweat samples, Lenochova et al. (2009) explored how repeated thawing cycles influence the perceived intensity, pleasantness, attractiveness, and masculinity of axillar sweat samples. The authors reported that only sweat intensity differed significantly from the first to the second thawing cycle. Nonetheless, the study did not rely on emotional sweat samples, and their conclusions are only based on subjective indicators (e.g., perceived intensity). In another study, de Groot et al. (2020), using a photoionization detector, quantified the volatile molecules in fear and neutral sweat samples across a first and a second application. The authors showed that the reused sweat released lower levels of volatiles when compared with its first use. However, despite the lower number of volatile molecules emitted, the authors did not examine the effects of the second-use sweat samples in communicating emotional information to their receivers. Thus, the information we have regarding the effectiveness of reusing an emotional sweat sample after it has been used once is sparse. The question that remains open is whether a second application of an emotional sweat sample with the same parameters as its first use elicits the same responses in its receivers as the first time it is used.

If a second use of the same sweat sample produces similar results, then this would offer a wide range of advantages aside from scales of economy regarding the cost of collecting sweat samples. Obviously, this would reduce costs and time, by a second use of the same samples. Additionally, this would also encourage replications by, for instance, other researchers who could be given access to the sweat samples used in an experiment.

Equally important as these advantages is the type of information one would be able to glean about the nature of the volatiles involved in the transmission of emotional information. It is known that high volatile molecules disperse faster and travel longer distances, with the clear advantage of carrying their “message” to different locations, however, for shorter time periods. In contrast, low-volatility molecules do not travel long distances. The information they carry remains for longer periods at the place of their emission. Consequently, the message they carry remains close to their location of emission, even when the sender is no longer there (e.g., Pause, 2017; Pause et al., 1997). Therefore, one may surmise that if the second use of odor samples does not give rise to the same cognitive, behavioral or psychophysiological reactions, then it is very likely that the message contained in the chemosignals is carried by high-volatility molecules. However, if their effects are comparable to those obtained in their first use, then one could infer that the message is likely transmitted through low-volatility molecules.

The main goal of the current study was to examine whether sweat samples collected in fear-inducing and neutral contexts would produce the same (or different) psychophysiological responses in a second application. To answer this question, we used the same sweat samples twice. The aim was to examine whether the facial electromyography (fEMG) effects obtained the first time would be reproduced with a second use of the sweat samples. Following previous research (e.g., de Groot et al., 2014), we expected that the exposure to fear sweat (compared with neutral sweat) would trigger a stronger activation of the *medial frontalis* and *corrugator supercilii*, at least in the first application of the sweat samples. Moreover, if the reuse of emotional sweat samples is a viable approach, then the fEMG activation patterns would be comparable across applications of the sweat samples. Notably, the question regarding the reuse of fear sweat samples was of an exploratory nature, relying on no strong a priori hypotheses regarding the outcome of the results.

## Method

### Sweat donors

#### Participants

Eight Caucasian Portuguese males aged 21–35 years (*M*_Age_ = 27.5 years; *SD* = 4.87) gave their informed consent and participated on a voluntary basis in two sweat collection sessions (fear- and neutral-inducing sessions), each separated by a week’s interval. Participants were heterosexual, nonsmokers, taking no medication at the time of the collection, and did not have any reported psychological or neurological disorders. Following previous guidelines regarding sweat collection (e.g., de Groot et al., 2015), only males were included as sweat donors because of their larger and more active apocrine glands than females (Zhou & Chen, 2009). Moreover, only heterosexual males were included as sweat donors because only female participants were recruited as sweat receivers (please see the sweat receivers section), and females seem to evaluate homosexual and heterosexual male sweat differently (Martins et al., 2005).

All the procedures for the sweat collection were approved by the host institution ethics committee and were conducted in accordance with the standards of the American Psychological Association and the guidelines of the Declaration of Helsinki.

#### Materials

##### Emotion induction film clips

In order to induce a fearful state or an unemotional (hereafter labeled “neutral”) state necessary for the sweat collection sessions, we selected, on the basis of a pilot study (*N* = 38), a set of short clips retrieved from horror movies (fearful condition) and several nature/animal-related documentaries and nature scenes (unemotional condition, which we label “neutral”) (for more information about the source of the film clips, see Appendix A). Participants rated their responses to the film clips on a 10-point visual analog scale, with the scale ends anchored as not at all (0) and very much (10). The results obtained in the pilot study revealed that participants exposed to the fearful clips (*N* = 20) reported significantly more fear (*M* = 6.63; *SD* = 3.62) than participants exposed to the neutral clips (*N = 18*; *M* = 1.03; *SD* = 1.70) [*t*(27.60) = 6.21; *p* < .001]. In line with this, participants exposed to the neutral clips also reported feeling significantly more neutral (*M* = 6.06; *SD* = 3.32) than participants exposed to the fear-inducing clips (*M* = 2.00; *SD* = 2.31) [*t*(36) = 4.42; *p* < .001].

##### Self-report questionnaires

Similar to the procedure employed by de Groot et al. (2015), sweat donors were asked to report, on separate 0–10 visual analog scales, to what extent they felt angry, fearful, happy, sad, disgusted, neutral, surprised, calm, and amused during the sweat collection session.

##### Sweat production calculation

Sweat was collected using nonwoven absorbent pads (70% viscose, 30% polyester; Wells, Sonae SA, Portugal). To determine the amount of sweat produced in each session, the pads were weighted using a Precisa scale (model: BJ 100M), with .001 g precision. The sweat production was calculated by subtracting the weight of the pad before the sweat collection session from the weight after the session.

#### Procedure

As in previous studies (e.g., de Groot et al., 2015; Kamiloğlu et al., 2018; Zhou & Chen, 2009), participants were required to follow a strict set of instructions during the 48 hours prior to each sweat collection session (i.e., fear-inducing and neutral sessions). This was done to prevent possible sources of odor contamination. In the two days that preceded each sweat collection, participants were instructed to shave their armpits and were not allowed to consume alcohol, have sexual intercourse, consume odorous food (e.g., garlic, chili, pepper, onion), practice excessive exercise, sleep in the same bed as their partner or pet, or use any type of perfume or perfumed deodorants. Participants received fragrance-free personal care products (i.e., soap, shampoo, and deodorant) and were only allowed to use these as their personal care products during those two days. On the collection day, participants were not allowed to wear any personal care products, even the deodorant that we provided them. Moreover, two hours before each sweat collection, participants were instructed not to eat or drink anything other than water.

Immediately before the sweat collection took place, participants were instructed to rinse their armpits and dry them with paper towels. Then the experimenter, wearing latex gloves and using hypoallergenic tape, attached the pads to the participants’ armpits. Before entering the collection room, participants were given a sterilized t-shirt and sweater which they had to wear during the collection. The temperature inside the collection room was kept between 23 and 25 °C.

Participants were then exposed to one of the two emotion-inducing film sets (i.e., fear or neutral). Each session lasted approximately 30 minutes. As in de Groot et al. (2015), film clips were presented from the least to the most intense to create a gradual buildup of emotional experience. At the end of each session, participants rated their feelings using separate 0–10 visual analog scales. Pads were then removed and frozen individually in Ambar vials at −80 °C.

Fear-inducing and neutral sweat collections were separated by a week’s interval. After completing the two collections, participants were debriefed and received monetary compensation.

#### Statistical analysis

Possible differences in room temperature and sweat production across the two sweat collection sessions (fear-inducing and neutral conditions) were examined using separate paired-samples Student *t*-tests.

As for the self-reported affect, and because the data did not present a normal distribution, separated nonparametric Wilcoxon tests were conducted to examine possible differences in these variables, across conditions.

The analyses were conducted using IBM SPSS statistical software (version 25.0; IBM Corp., Armonk, NY).

### Sweat receivers

#### Participants

Sixty-four female university students gave their informed consent and participated in the experiment on a voluntary basis. Four participants were excluded from the experiment due to psychiatric disorders, ethnic background other than Caucasian, and a software error that resulted in the loss of data. Thus, 30 participants from Utrecht University (the Netherlands), aged 19–34 years (*M*_Age_ = 23.20 years; *SD* = 3.11), took part in the first sweat-sample use, and 30 participants from ISPA – Instituto Universitário (Portugal), aged 19–35 years (*M*_Age_ = 23.93 years; *SD* = 5.32), were in the second-use sweat-sample experiment. All participants were right-handed, Caucasian, and nonsmokers, who reported no psychiatric or neurological disorders, no respiratory disease, and no illness, cold, or allergy. Moreover, participants were screened for severe olfactory impairments (i.e., anosmia). All participants appeared not to suffer from anosmia since they were able to clearly identify three odors: cinnamon, fish odor, and banana (see Lötsch et al., 2016).

Only females were recruited due to their higher sensitivity towards emotional signals and a better sense of smell compared to men (see de Groot et al., 2015; Zhou & Chen, 2009). Moreover, research has shown that women perceive male sweat differently as a function of both the donors’ and their own sexual orientation (Martins et al., 2005). Therefore, only heterosexual women were included as sweat receivers.

Both the first and the second sweat-sample studies were approved by the host institutions ethics committees (Utrecht university and ISPA, respectively), and were conducted in accordance with American Psychological Association standards and the guidelines of the Declaration of Helsinki.

#### Design

The present study employed a 2 × 2 design, with two sweat conditions (fear vs. neutral; within-subject factor) for each of two sweat applications (first vs. second use; between-subject factor). Sweat conditions were presented in a counterbalanced order, and neither the participants nor the experimenter were aware of the conditions (i.e., double-blind experiment). Moreover, the first and second sweat applications were separated by an interval of approximately one year.

#### Materials and measures

##### Composition of sweat stimuli

As in previous studies (e.g., de Groot et al., 2015; Kamiloğlu et al., 2018), in order to reduce the effects of interindividual variability in sweat production, pad pieces from four sweat donors were combined to create a super-donor. While still frozen, each pad obtained from the sweat donors’ armpits was cut into eight equal parts. Using a randomization script, each final “super-donor” sample consisted of four pad pieces (two from the left and two from the right armpits), collected from four distinct sweat donors. Each sweat receiver was exposed to the same combination of sweat donors across the two sweat conditions. The “super-donor” samples were prepared and coded by an independent researcher. Thus, the experimenter was completely blind to the sweat conditions during the experiment.

##### Facial electromyography (fEMG)

Ag-AgCl EMG electrodes were applied in bipolar fashion to the left *corrugator supercilii* and *medial frontalis*, two muscles involved in displaying fearful facial expressions (see Fridlund & Cacioppo, 1986). Only the left facial side was monitored, as this side seems to display stronger affective reactions than the right side in right-handed participants (see Dimberg & Petterson, 2000). The goal of the fEMG responses was to compare the activity of the two muscles across the two sweat conditions in the two sweat applications.

The EMG signal was collected using a BioNex eight-channel chassis, powered by BioLab software (first sweat application: version: 3.0.0; second sweat application: version 3.2.0; MindWare Technologies, Gahanna, OH). During the data collection, the signal was online-filtered using a 20–200 Hz bandpass filter. Before analyzing the data, the fEMG signal was also rectified and smoothed with a 20 Hz low-pass filter using EMG Analysis software (version 3.1.5 for both sweat applications; MindWare Technologies, Gahanna, OH).

##### Handedness questionnaire

In order to control for possible effects of handedness on EMG data (see Dimberg & Petterson, 2000), as well as to confirm that all participants were, in fact, right-handed, a handedness questionnaire (see Williams, 1986) was used.

##### Sweat ratings

Participants were asked to rate, in a counterbalanced order, the hedonic value (pleasantness) and intensity of the sweat samples that they were exposed to during the experiment on seven-point Likert scales. The scale ends were anchored with “very weak” (1) and “very strong” (7) in the case of intensity, and with “very unpleasant” (1) and “very pleasant” (7) in the case of pleasantness.

##### Sweat discrimination

Participants performed a two-alternative forced-choice reminder task (de Groot et al., 2014) to evaluate their ability to discriminate between sweat sample conditions (fear and neutral) that were used in the experiment.

#### Procedure

**Fig. 1:**
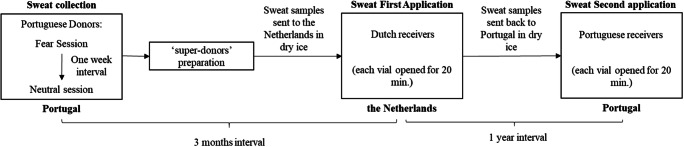
Flowchart of the general experimental procedure, from the sweat collection to the first and then the second sweat application. The respective time intervals between the different phases, as well as the countries where they occurred, are identified

The procedure was replicated for the two odor uses, with the odor conserved from the first to the second use at −80 °C in amber vials. The sweat samples were transported from Portugal to the Netherlands, and then back to Portugal in dry ice. The first and the second sweat applications were separated by an interval of a year (see Fig. 1 for a general flowchart of the procedure). Moreover, all data collection sessions were conducted by female experimenters to avoid mood changes in the female participants that the presence of a male experimenter could induce (see Jacob et al., 2001).

Each data collection session began by thawing the sweat samples an hour and a half prior to the start of the experiment. After entering the lab, participants were given brief instructions about the experiment and the task, plus the fEMG devices that would be used. Participants were then instructed to complete a sociodemographic questionnaire with some personal information. The experimenter began by cleaning the skin on the left side of the participant’s face and applying fEMG electrodes on the *medial frontalis* and *corrugator supercilii* muscles. Following the fEMG setup, participants were given instructions via a computerized task, similar to those used by Kamiloğlu and colleagues (2018). This was for replication purposes, and the collected data were unrelated to the goal of the present study, our main focus being the fEMG activity. Next, participants filled out a handedness questionnaire while the experimenter put the first vial (containing either a fear or a neutral sweat sample in a counterbalanced order) in a vial holder (flexible claw). Then participants were instructed to place their head on a chin rest, and the vial holder was adjusted such that the vial with the sweat sample was 2 cm below the participant’s nostrils. After a brief practice phase that allowed participants to become familiar with the task, and before starting the experimental task, the participants’ nostrils were closed with a nose clip and they were told to direct their gaze at the fixation cross on the screen. The vial with the sweat samples was opened and immediately after starting the experimental task, the experimenter also removed the nose clip. The experiment began with a fixation cross that remained on the screen for 5 seconds, and then the computerized task proceeded. When the first block was completed, the experimenter changed the vial and placed the second vial containing either a fear- or a neutral-related sweat sample (counterbalanced). After a mandatory 5-minute break, the procedure for the experimental task was repeated for the remaining odor condition. At the end of the task (two blocks), the fEMG electrodes were removed, and the participants were asked to rate the hedonic value (pleasantness) and intensity of the sweat samples. The absence of severe olfactory impairments in participants (Lötsch et al., 2016) and their capacity to discriminate between sweat conditions were then assessed. At the end of the experimental procedure, participants were debriefed about the study’s main goals and received monetary compensation.

Each data collection session lasted 60 minutes, comprising 15 minutes of facial preparation and fEMG electrode placement, and 40 minutes of experiment, with a mandatory 5-minute pause between sweat conditions. Each vial remained opened for 20 minutes.

#### Data preparation

Although the fEMG signal was continuously collected during the experiment, only the first 4.6 seconds after sweat exposure was extracted and analyzed. First, the fEMG data were checked for artifacts in intervals of 50 ms. For each participant, each muscle and odor condition, values higher than 2.5 median absolute deviation (MAD) units (Leys et al., 2013) were marked as artifacts. Then, using participants’ facial video recordings, the identified artifacts were visually inspected to ensure that they were associated with a non-odor-related movement (e.g., sneezing). If such was observed, then these artifacts were removed from the signal, otherwise, they remained untouched. Missing data removed due to artifacts were linearly interpolated, using the R package “*Zoo*” (Zeileis & Grothendieck, 2005) (for information regarding the mean percentage of interpolated data per participant, see Appendix B).

Following earlier studies (e.g., Kamiloğlu et al., 2018), fEMG data were then averaged in 200 ms intervals: the first three intervals (600 ms) constituted the baseline (since the typical first sniff starts at around 400 ms; see Kamiloğlu et al., 2018; Sela & Sobel, 2010); the remaining 20 intervals (4 seconds) constituted the target signal. In sum, the first 600 ms of collected signal represented the baseline period and the next 4 second the test period. As in previous studies (e.g., Kamiloğlu et al., 2018), prior to the analysis, fEMG data were screened for outliers (within variable, i.e., the 23 200-ms intervals), defined as values exceeding (below or above) 2.5 median absolute deviation (MAD) units (Leys et al., 2013). Participants with percentages of outlier data above 75% were excluded from the final analysis [(number of excluded participants in the sweat first use: fear condition *medial frontalis* = 1; fear condition *corrugator supercilii* = 3; neutral condition *medial frontalis* = 2; neutral condition *corrugator supercilii =* 3); (number of excluded participants in the sweat second use: fear condition *medial frontalis =* 4; fear condition *corrugator supercilii* = 2; neutral condition *medial frontalis* = 4; neutral condition *corrugator supercilii =* 2)]. As in previous studies (e.g., Kamiloğlu et al., 2018), missing data due to outlier-based removal were altered to be one unit above the next extreme score on that variable (see Field, 2013) (for more information regarding the mean percentage of altered data per participant, see Appendix C).

The fEMG data analysis was based on baseline-corrected data, obtained by subtracting from each 200-ms segment the mean activity of the corresponding muscle’s baseline.

#### Statistical analysis

Regarding the fEMG data^1^, our aim was to examine whether exposure to fear sweat samples (compared to neutral sweat samples) induces higher activity in the facial muscles involved in fearful facial expressions (i.e., *the medial frontalis* and the *corrugator supercilii*), in both the first and second use of the same sweat samples. Thus – after a visual inspection of the residual plots that did not reveal any severe violation of the homoscedasticity or normality assumptions – two separate linear mixed models (LMM; one for each muscle) were conducted including the subjects ID as a clustering factor, the muscle activation as the dependent variable, linear and quadratic time (i.e., 20 200-ms time intervals) as continuous independent variables, and the sweat application (first vs. second use; between subjects) and the sweat condition (i.e., fear vs. neutral sweat samples; within subjects) as predictors to the model. In order to estimate the linear trend of time and to ease the parameter interpretation, the variable time was centered. As fixed effects in the model, we consider the sweat use, the sweat condition, the linear and quadratic time, as well as their two-way and three-way interactions. The quadratic effect of time was considered because (a) a visual inspection suggested that a quadratic trend provided a better fit to the data pattern, which had also been seen in earlier studies (see, for instance, Kamiloğlu et al., 2018; Fig. 3), and (b) a combination of linear and quadratic effects of time allowed us uncover not only the general increment of the muscle activation over time (i.e., the linear effect) but also the pattern of this increment (i.e., the quadratic effect). As the quadratic effect of time proved to be significant (for both the *medial frontalis* and *corrugator supercilii*), it was retained in the models.

As random effects, we considered random intercepts per subject, as well as by subject random slopes for the sweat condition, linear time, and their interaction. Following Little and colleagues (2000), any parameter with variance greater than 0 was left as random. Moreover, the model was estimated using restricted maximum likelihood, and a Satterthwaite approximation of the degrees of freedom was considered (see West, 2009). The LMM analyses were performed using the GAMLj module (Gallucci, 2019) implemented with the jamovi software (The jamovi project, 2019).

To examine possible differences in the perceived hedonic value (pleasantness) and intensity of the sweat samples, two separate 2 × 2 mixed factorial ANOVA designs (one for the intensity and the other for the pleasantness) were carried out. Sweat condition (fear vs. neutral sweat) was entered as the within-subject factor, and sweat application (first vs. second use) was entered as the between-subject factor. Finally, one-sample Wilcoxon signed-rank tests were used to evaluate whether the participants were able to discriminate between sweat conditions. These analyses were conducted using IBM SPSS statistical software (version 25.0; IBM Corp., Armonk, NY).

## Results

### Sweat donors

**Fig. 2:**
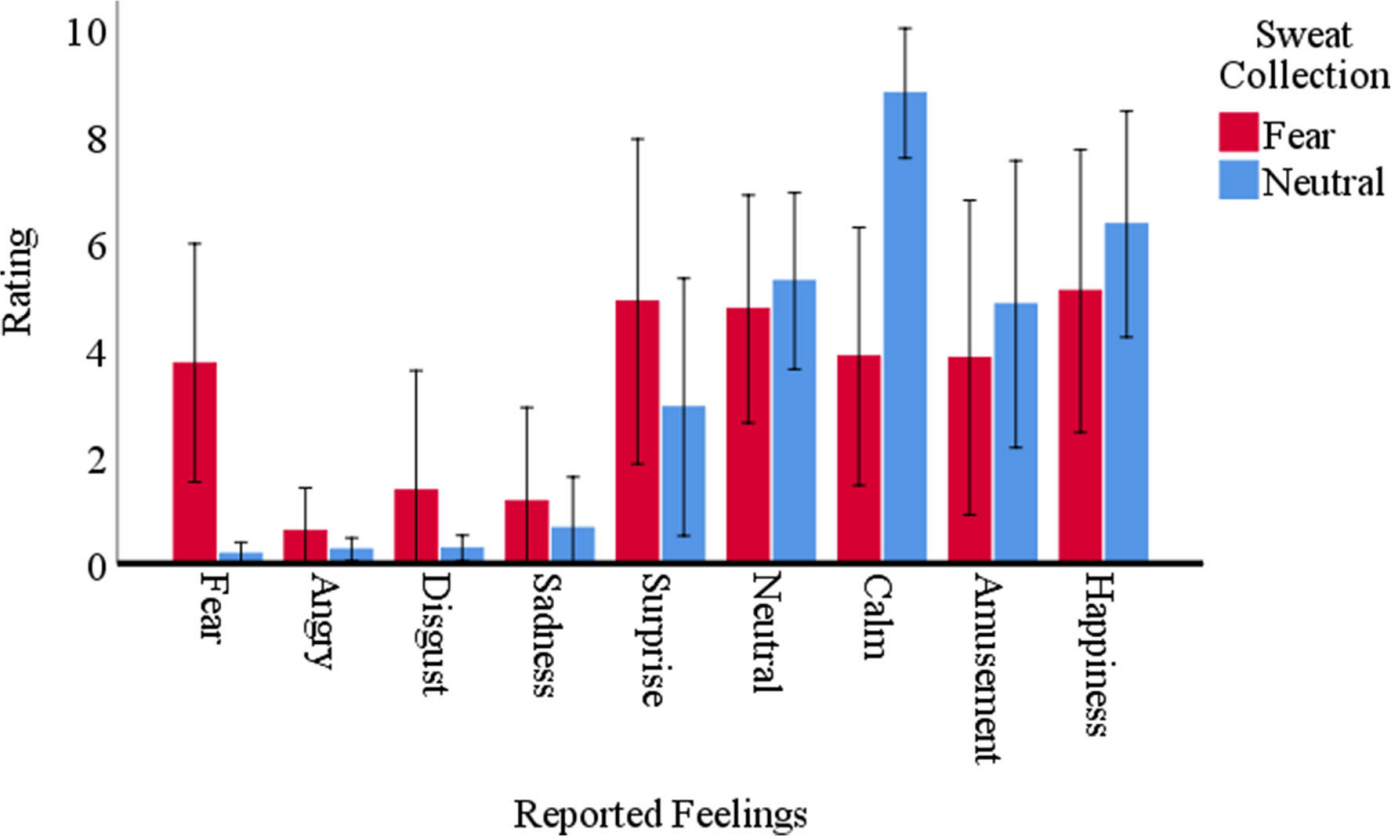
Mean reported feelings by sweat donors, per sweat collection. Error bars represent 95% within-subject confidence intervals

Concerning the self-reported questionnaires, the nonparametric Wilcoxon signed-rank test revealed that, as expected, participants reported more fear (*N = 8*; *Z* = −2.37; *p* =.016) in the fear condition (*Mdn* = 3.60; *IQR* = 2.15 – 5.2) than in neutral condition (*Mdn* = .05; *IQR* = .00 – 0.35). Regarding calmness, more calmness (*N = 8*; *Z* = −2.37; *p* =.016) was reported in the neutral condition (*Mdn* = 9.60; *IQR* = 7.25 – 10.00) than the fear condition (*Mdn* = 3.65; *IQR* = 1.85 – 4.68), pointing to a successful emotional manipulation during the sweat collection sessions. Furthermore, the results showed statistically significant differences in reported happiness (*N = 8*; *Z* = −2.20; *p* =.031), with more happiness reported in the neutral condition (*Mdn* = 6.35; *IQR* = 4.98 – 7.98) than in the fear condition (*Mdn* = 5.50; *IQR* = 3.93 – 6.75). Surprisingly, no statistically significant differences were observed in the reported neutral affect between the fear (*Mdn* = 4.85; *IQR* = 4.10 – 5.13) and the neutral (*Mdn* = 5.00; *IQR* = 4.63 – 5.20) conditions (*N = 8*; *Z* = −.28; *p* = .811). Moreover, no statistically significant differences were observed between conditions in the reported anger (*N = 8*; *Z* = −.73; *p* = .625), disgust (*N = 8*; *Z* = −1.75; *p* = .130), sadness (*N = 8*; *Z* = −1.38; *p* = .203), surprise (*N = 8*; *Z* = −1.02; *p* =.359), and amusement (*N = 8*; *Z* = −1.01; *p* =.380). Figure 2 provides an overview of the self-reported affect.

**Fig. 3:**
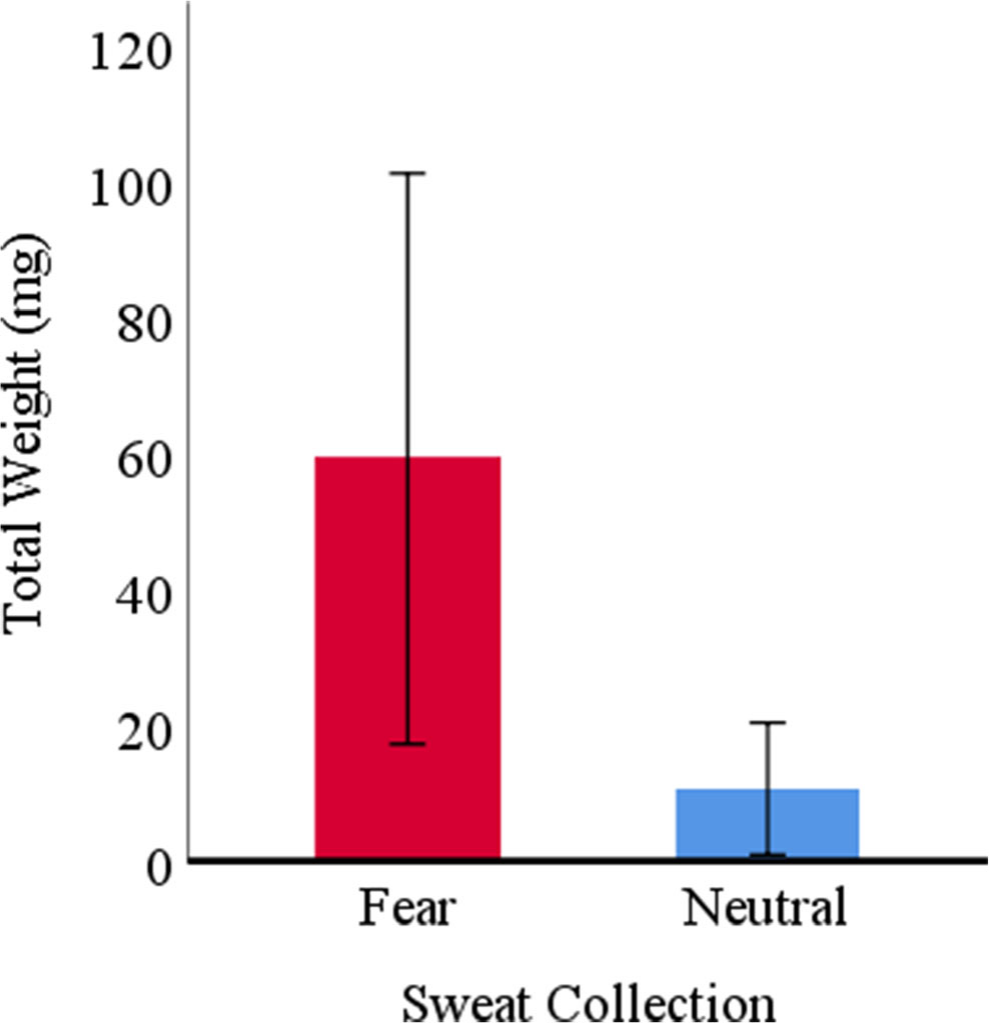
Mean sweat production, in milligrams, per sweat collection. Error bars represent 95% within-subject confidence intervals

Regarding sweat production, a paired-samples Student *t*-test revealed that participants in the fear condition (*M* = .06 g; *SD* = .05) produced significantly more sweat [*t*(7) = 2.56; *p* = .038] than those in the neutral condition (*M* = .01 g; *SD* = .01) (see Fig. 3), suggesting that the emotional manipulation influenced participants’ sweat production. Additionally, with regard to room temperature, a paired-samples Student *t*-test revealed no statistically significant differences [t(7) = 1.16; p = .285] between the fear (*M* = 24.05; *SD* = .76) and the neutral conditions (*M* = 24.13; *SD* = .64), ruling out the role of temperature in the differences observed in sweat production.

Taken together, the results obtained from the sweat donors (*N* = 8) suggest successful emotion manipulation during sweat collection. Although the subjective fear ratings in the fear condition are low, and do not exceed the ratings of neutral emotion, calmness, surprise, amusement, and happiness (as can be seen in Fig. 2), the fear rating remains significantly higher than the fear ratings for the neutral condition. Additionally, the differences observed in sweat production – an objective measure – show that the emotional manipulation modulated participants’ perspiration. In line with previous research (see de Groot et al., 2015) more sweat was produced during the fear than the neutral condition. The observed low fear rating might be explained by a social desirability factor: for cultural reasons, men tend to report lower levels of fear (e.g., Spiegler & Liebert, 1970). However, as no social desirability measure was used, no strong conclusions can be drawn.

### Sweat receivers

#### fEMG

##### Medial frontalis muscle

**Fig. 4:**
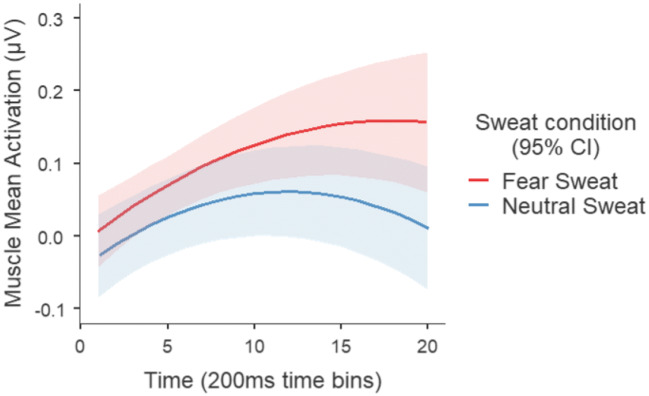
Mean activation of the medial frontalis in microvolts (μV), per sweat condition, across sweat applications. Each time point represents a 200 ms time bin. The shaded area represents 95 % confidence intervals

The LMM analysis (*R*^*2*^_*marginal*_ = .05; *R*^*2*^_*conditional*_ = .60) revealed a significant main effect of sweat condition (*B* = −.07; *F*(1, 60.9) = 4.89; *p* = .031; *95% CI* [−.131; −.008]), suggesting that, overall, the fear sweat (*M* = .13 μV; *SE* = .03) activates the *medial frontalis* muscle more than the neutral sweat (*M* = .06 μV; *SE* = .03). Moreover, a significant interaction was also revealed between sweat condition and linear time (*B* = −.01; *F*(1, 53.3) = 4.24; *p* =.044; *95% CI* [−.012; −2.88*10^−4^]), indicating that the activation of the medial frontalis diverges between sweat conditions over time for the two sweat applications (see Fig. 4). These results are in accordance with previous studies (e.g., de Groot et al., 2012, 2014, 2015; Kamiloğlu et al., 2018), showing that the exposure to sweat produced under fear states results in higher activation (compared with the exposure to neutral sweat) of the *medial frontalis*, one of the facial muscles involved in fear facial expression (see Fridlund & Cacioppo, 1986).

Notably, the LMM showed no main effect of sweat application (*B* = .03; *F*(1, 57.7) = .34; *p* = .560; *95% CI* [−.065; .121]), no interaction between sweat application and sweat condition (*B* = .03; *F*(1, 60.9) = .30; *p* = .583; *95% CI* [−.088; .158]), no interaction between sweat application, sweat condition, and linear time (*B* = 4.64*10^−3^; *F*(1, 53.3) = .65; *p* = .425; *95% CI* [−.007; .016])], and no interaction between sweat application, sweat condition, and quadratic time (*B* = −4.72*10^−4^; *F*(1, 1948.4) = .74; *p* = .390; *95% CI* [−.002; 6.05*10^−4^]). These results indicate that there are no significant differences between the first and second use of the sweat samples, pointing to the reliability of reusing sweat samples, at least a second time^2^.

Additionally, although not relevant to test our hypothesis, there was also a significant main effect of linear (*B* = 4.91*10^−3^; *F*(1, 53.9) = 7.23; *p* = .010; *95% CI* [.001; .008]) and quadratic time (*B* = −6.37*10^−4^; *F*(1, 1948.4) = 21.51; *p* < .001; *95% CI* [−9.06*10^−4^; −3.68*10^−4^]), and a significant interaction between sweat application and linear time (*B* = .01; *F*(1, 53.9) = 12.41; *p* < .001; *95% CI* [.006; .020]). Moreover, there was no interaction between sweat application and quadratic time (*B* = 5.23*10^−4^; *F*(1, 1948.4) = 3.62; *p* = .057; *95% CI* [−1.59*10^−5^; .001]), and no interaction between sweat condition and quadratic time (*B* = −2.25*10^−4^; *F*(1, 1948.4) = .67; *p* = .412; *95% CI* [−7.64*10^−4^; 3.13*10^−4^]).

##### Corrugator supercilii muscle

**Fig. 5:**
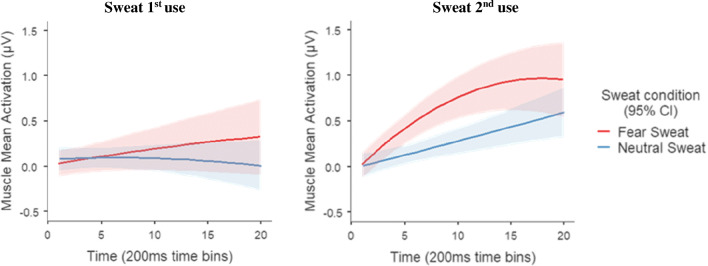
Mean activation of the corrugator supercilii in microvolts (μV), per sweat condition and sweat application. Each time point represents a 200 ms time bin. The shaded area represents 95 % confidence intervals

The LMM analysis (*R*^*2*^_*marginal*_ = .15; *R*^*2*^_*conditional*_ = .78) revealed a significant main effect of sweat condition (*B* = −.30; *F*(1, 55.7) = 10.23; *p* = .002; *95% CI* [−.484; −.116]), with overall stronger activation of this muscle when participants were exposed to fear (*M* = .49 μV; *SE* = .09) than to neutral sweat (*M* = .19 μV; *SE* = .05). Furthermore, a significant interaction between sweat condition and linear time was also found (*B* = −.02; *F*(1, 54.7) = 6.45; *p* = .014; *95% CI* [−.034; −.004]), suggesting that the activation of the *corrugator supercilii* diverges between sweat conditions, over time, across sweat applications (see Fig. 5). Once again, and in accordance with previous studies (e.g., de Groot et al., 2014; Kamiloğlu et al., 2018), these results showed that the exposure to fear sweat (compared to the exposure to neutral sweat) results in a stronger activation of the *corrugator supercilii* – another muscle related with the facial expression of fear (see Fridlund & Cacioppo, 1986).

Moreover, although a significant main effect of sweat application was revealed (*B* = .40; *F*(1, 57.2) = 13.13; *p* < .001; *95% CI* [.182; .609]), there was no significant interaction between sweat condition and sweat application (*B* = −.37; *F*(1, 55.7) = 3.97; *p* = .051; *95% CI* [−.742; −.006]), or between sweat application, sweat condition, and linear time (*B* = 1.07*10^−3^; *F*(1, 54.7) = .01; *p* = .943; *95% CI* [−.028; .030]). These results seems to indicate that, even though the two sweat applications had different overall activations [first use: *M* = .14 μV; *SE* = .08; second use: *M* = .54 μV; *SE* = .08], the linear data trend did not differ across the two sweat use conditions, suggesting, once again, that the use of sweat samples a second time is reliable^3^. However, a significant interaction was shown between sweat application, sweat condition, and quadratic time (*B* = 3.56*10^−3^; *F*(1, 1976) = 12.57; *p* < .001; *95% CI* [.002; .006]), indicating that the quadratic data trend may vary between sweat applications (see Fig. 4). Possible explanations for the observed differences in the corrugator activity between sweat applications are explored in the discussion section.

Furthermore, and again not directed towards our hypotheses, we observed a significant main effect of linear (*B* = .02; *F*(1, 56.4) = 20.76; *p* < .001; *95% CI* [.013; .033]) and quadratic time (*B* = −9.90*10^−4^; *F*(1, 1976) = 15.53; *p* < .001; *95% CI* [−.001; −4.98 e-4]), and significant interactions between sweat condition and quadratic time (*B* = 1.55*10^−3^; *F*(1, 1976) = 9.48; *p* = .002; *95% CI* [5.63*10^−4^; .003]), sweat application and linear time (*B* = .03; *F*(1, 56.4) = 11.66; *p* < .001; *95% CI* [.015; .054]), and sweat application and quadratic time (*B* = −1.23*10^−3^; *F*(1, 1976) = 6.01; *p* = .014; *95% CI* [−.002; −2.47*10^−4^]).

#### Sweat ratings

Concerning the perceived intensity of sweat, the results showed no significant main effect for the sweat condition [*F*(1, 58) = .87; *p* = .354] or sweat use condition [*F*(1, 58) = 3.60; *p* = .063], and no interaction between sweat condition and sweat use [*F*(1, 58) = .02; *p* = .877]. In sum, these results indicate that, participants did not perceive a difference in sweat intensity across sweat conditions or sweat administration.

Similarly, regarding perceived pleasantness the results revealed no significant main effect of the sweat condition [*F*(1, 58) = .04; *p* = .842], and no significant interaction between the sweat application and the sweat condition [*F*(1, 58) = .64; *p* = .427]. However, a main effect was found for the sweat application [*F*(1, 58) = 17.63; *p* = .018] , with the sweat samples – regardless of the sweat condition – rated as more pleasant in the second (*M* = 4.32; *SE* = .22) than in the first use (*M* = 3.55; *SE* = .22). Thus, these results show that no difference in pleasantness was consciously perceived between sweat conditions in either sweat use condition. However, from the first to the second use, the perceived pleasantness seems to have increased (see Table 1).

**Table 1:** Means and standard deviations (in parenthesis) of the subjective ratings of the sweat stimuli

	Fear sweat				Neutral sweat			
	First application		Second application		First application		Second application	
Subjective ratings of stimuli								
Intensity (1 = very weak to 7 = very strong)	2.90	(1.30)	2.37	(1.67)	2.73	(1.46)	2.13	(1.22)
Pleasantness (1 = very unpleasant to 7 = very pleasant)	3.60	(1.30)	4.23	(1.50)	3.50	(1.11)	4.40	(1.57)

Moreover, when both intensity and pleasantness were entered into the two separate LMMs as control covariates, the interpretation of the results remained the same. Table 2 provides an overview of the principal main effects and interactions after entering the covariates in the model.

**Table 2:** Principal main effects and interactions after entering intensity and pleasantness as covariates in the LMMs

	Medial frontalis			Corrugator supercilii		
	*B*	*F*	*p*	*B*	*F*	*p*
Effect						
Sweat application	.02	.14	.707	.42	14.60	< .001
Sweat condition	−.07	5.26	.025	−.30	10.04	.003
Sweat condition*Time	−.01	4.28	.043	−.02	6.40	.014
Sweat application*Sweat condition	.03	.25	.618	−.37	3.93	.052
Sweat application*Sweat condition*Time	4.62*10^−3^	.64	.428	7.74*10^−4^	2.68*10^−3^	.959
Sweat application*Sweat condition*Time^2^	−4.72*10^−4^	.74	.391	3.56*10^−3^	12.57	< .001

#### Sweat discrimination

Regarding the first sweat use, a one-sample Wilcoxon signed-rank test revealed that participants were successful in discriminating both the neutral (from the fear) sweat when this was presented as reference sweat (*median under the null hypothesis* = .50; *N* = 30; *Z* = 2.67, *p* = .008), and the fear (from the neutral) sweat when fear sweat was presented as reference (*median under the null hypothesis* = .50; *N* = 30; *Z* = 2.00, *p* = .046).

As far as the second sweat application is concerned, the results also showed that participants were able to discriminate the neutral (from the fear) sweat sample (when presented as reference)(*median under the null hypothesis* = .50; *N* = 30; *Z*= 2.67, *p* = .008), but not the fear (from the neutral) sweat sample (when this sample was presented as reference) (*median under the null hypothesis* = .50; *N* = 30; *Z* = .89, *p* = .371).

## Discussion

The aim of the research reported in this paper was to test whether sweat samples (i.e., fear and neutral) obtained from donors can be reliably used on two separate occasions. We compared psychophysiological responses (i.e., fEMG) obtained across two administrations of the same odors (fear vs neutral) one year apart. The results of the study showed that in both the first and the second administration of the sweat samples, the exposure to fear sweat (compared to neutral sweat) triggered a significantly higher activation of the facial muscles involved in the fear facial expression (i.e., the *medial frontalis* and the *corrugator supercilii*; Fridlund & Cacioppo, 1986). Furthermore, the perceived intensity and pleasantness between the sweat samples revealed no differences within each of the two applications of the sweat samples, ruling out the possibility that either dimension could have contributed to the observed distinct fEMG activation patterns between the fear and neutral sweat conditions (e.g., Kamiloğlu et al., 2018). This set of results shows that the reuse of sweat samples collected in emotion contexts is reliable, at least a second time.

The conclusions presented here have a number of relevant implications for research on human chemosignals produced under emotional conditions. The ability to use the same sweat sample twice (1) reduces the amount of sweat required to run a study, (2) reduces cost, (3) facilitates possible replication studies (even across labs), and (4) allows researchers to conduct studies with larger sample sizes, thus limiting potential criticisms of sample sizes used in research with chemosignals (for a similar argument see Lenochova et al., 2009).

The robustness of reusing sweat samples becomes even more remarkable when one considers that the sweat samples were collected from Portuguese donors and then sent to the Netherlands where they were first used. In this case, the receivers were Dutch. The sweat samples were then sent back to Portugal, where they remained frozen at −80 °C for a year before the second application took place. This time, however, the receivers were Portuguese (see methods section). The second application produced comparable fEMG findings, suggesting that the emotional information carriers in the sweat were not lost, despite a year’s interval between the two applications.

The only difference between the first and second applications was observed in the case of the *corrugator supercilii* activity. There was an overall stronger muscle activation in the second versus the first sweat application, along with a distinct quadratic time data trend. Thus, although the data pattern was similar, some differences were observed between the two applications. It is difficult to interpret this difference, since there are multiple possible contributors to the observed difference between the two applications. The equipment was identical between studies. We could speculate that the difference between the first and second applications resulted from potential cultural differences between sweat donors and receivers. It is hypothetically possible that Portuguese persons frown more in general, and thus sweat samples showed a stronger *corrugator supercilii* response in the Portuguese sample than the Dutch sample. It is also possible that the odor of sweat from Portuguese donors is more familiar to Portuguese than to Dutch receivers, which might have increased the pleasantness ratings of the sweat (for a relation between repeated exposure to odor and increased pleasantness see Delplanque et al., 2009) and also the *corrugator supercilii* activity. But this remains mere speculation. On the other hand, and considering that the sweat samples (regardless of the sweat condition) were rated as more pleasant in the second than in the first application, it is possible that the reuse of the samples, their shipping, and/or the one-year storage may have changed some of the chemical properties of the samples. Consequently, these changes may have induced the stronger *corrugator supercilii* activation observed in their second application. Hence, it is important to note that we are not claiming that our results show that the chemical composition of the sweat samples remained unchanged between applications. Instead, our argument is that, despite possible chemical changes in the sweat samples, the chemicals responsible for the fear signal were preserved across applications.

The findings of our research also provide some cues about the properties of the emotional sweat samples. To date, few attempts have been made to unravel the chemical properties of human sweat (e.g., Penn et al., 2007; Smeets et al., 2020). Consequently, what we know about sweat volatiles that carry emotional information is limited. In a recent study, de Groot et al. (2020) demonstrated that fear sweat emits a higher quantity of volatiles than neutral sweat. However, the quantity of volatiles emitted decreased significantly in a second application of the same sweat samples (see de Groot et al., 2020). The data from the aforementioned study, together with our results, seem to suggest that although the quantity of volatiles is reduced in a second application of the same sweat samples, the chemical compounds communicating fear information are preserved. Moreover, as we mentioned in the introduction, high-volatility molecules are known to spread faster than low-volatility molecules, conveying their information to distant locations. In contrast, low-volatility molecules remain at the same location for longer periods, retaining their “message” (see Pause, 2017; Pause et al., 1997). The fact that the two emotional sweat sample applications induced the same psychophysiological reactions across time (one year) and location (Utrecht and Lisbon) indicates that the volatiles preserved the relevant information. This suggests that low-volatility molecules are more likely the carriers of emotional information. It is then hypothetically possible that the decrease in the quantity of volatiles from a first to a second application of the same sweat samples, as observed by de Groot et al. (2020), is associated with high-volatility molecules – which are likely to be the first to dissipate. The molecules that remained from the first to the second sweat application – i.e., the low-volatility compounds – may have continued to carry the fear-related information to their receivers, resulting in the comparable fEMG activation patterns observed in our study.

Nevertheless, it is important not to forget that de Groot et al. (2020) quantified the sweat volatiles that were released by a continuous-flow olfactometer, which is inherently different from the sweat delivery method used in our study, in which sweat was sampled with pads which were then put in vials and presented to receiver participants. It is, therefore, possible that the different delivery methodologies affected the outcomes of the studies: the continuous airflow in the olfactometer in de Groot et al. (2020) could have diluted the sweat stimulus to a greater extent than the delivery method used in our study, reducing the quantity of volatiles available in the second application. Further research is required to assess whether our findings generalize to other delivery methods (e.g., an olfactometer; Lundström et al., 2010).

A final consideration that might prove relevant and perhaps limit the scope of our conclusions is that our senders and receivers were males and females, respectively. Although this procedure is common to most studies (e.g., de Groot et al., 2012, 2014, 2015; Kamiloğlu et al., 2018), it is nonetheless a variable that needs to be addressed.

Lastly, Smeets et al. (2020) recently took the first step in uncovering the chemical fingerprint of fear sweat. In their work, the authors showed that fear and neutral sweat have distinct chemical signatures, providing a list of candidate chemical classes associated with emotional sweat. Considering our results, together with the findings from de Groot et al. (2020), future chemical analyses could benefit from using sweat after it has been used once, as compounds that are not strictly necessary to communicate fear through olfaction may dissipate in a first application of the sweat samples, reducing the noise in chemical analyses using a second application of sweat samples.

In conclusion, we have been able to show that the reuse of fear (and neutral) sweat samples is feasible, as they induce similar fEMG activity in their receivers across a first and second application. To our knowledge, this is the first study that approaches this issue, which aside from practical implications for future research with human chemosignals, raises potentially interesting questions regarding the chemical properties of emotion-inducing human odors. Are the carriers of emotion-related information in human odors high or low volatiles? The tentative direction our research suggests is that low volatiles carry emotion-relevant information.

## Appendix A. Information about the source of the selected film clips

For the fear-inducing condition, the clips were selected from the following terror films: *The Nun* (04 min 54 s), *Mamma* (07 min 40 s), *Sinister* (02 min 07 s), *The Descent* (02 min 41 s), *The Grudge* (02 min 10 s), *REC 1* (02 min 53 s), *Insidious* (04 min 55 s), and *A Tale of Two Sisters* (07 min 30 s).

Regarding the neutral condition, the clips were part of the documentaries *Solar eclipse* (02 min 37 s), *The Secret Life of Birds* (04 min 25 s), *The Transit of Venus* (03 min 02 s), *Equator: Battle for the light* (02 min 12 s), *Do we need the moon?* (02 min 09 s), *Discovery decade* (01 min 42 s), *Portugal Earth* (03 min 08 s) and *Wooly mammoth* (03 min 36 s), and nature sceneries retrieved from YouTube (11 min 40 s).

## Appendix B. Mean percentage of interpolated data per participant

Sweat first use: fear condition *medial frontalis* = 4.07%; fear condition *corrugator supercilii* = 6.83%; neutral condition *medial frontalis* = 6.27%; neutral condition *corrugator supercilii* = 6.37%.

Sweat second use: fear condition *medial frontalis* = 4.02%; fear condition *corrugator supercilii* = 6.01%; neutral condition *medial frontalis* = 4.96%; neutral condition *corrugator supercilii* = 6.38%.

## Appendix C. Mean percentage of altered outlier data per participant

Sweat first use: fear condition *medial frontalis* = 10.34%; fear condition *corrugator supercilii* = 3.56%; neutral condition *medial frontalis* = 9.14%; neutral condition *corrugator supercilii* = 5.19%.

Sweat second use: fear condition *medial frontalis* = 11.71%; fear condition *corrugator supercilii* = 11.18%; neutral condition *medial frontalis* = 7.86%; neutral condition *corrugator supercilii* = 9.63%.

## Footnotes

1 Regarding the fEMG activity, we changed the preregistered analysis plan. Instead of the planned repeated-measures ANOVAs, we decided to use linear mixed models, which have been demonstrably advantageous in analysing physiological data (e.g., Wolfinger, 1997). These advantages include, for instance, the possibility to treat time as a continuous variable, add a quadratic description of nonlinear changes over time, and also to deal with individual variability across time (see Krueger & Tian, 2004).

2 To further support the observed nonsignificant differences between sweat applications, a log-likelihood ratio test (LRT) comparing the reported model with a model without the three-way interactions was conducted, and the variance explained by each model was inspected (*R*^*2*^_*Conditional*_*)*. The LRT revealed that no significant differences between the two models [*X*^2^(2) = 1.41, *p* = .495], and the *R*^*2*^_*Conditional*_ for the two models was the same: .60. Hence, the variance explained by the two three-way interactions is apparently very small, providing further evidence – beyond the already reported *p*-values – for the absence of differences between sweat applications.

3 Once again, to further support the observed nonsignificant differences between sweat applications, a log-likelihood ratio test (LRT) comparing the reported model with a model without the nonsignificant three-way interaction was conducted, and the variance explained by each model was inspected (R^2^_Conditional_). The LRT test revealed no significant differences between the models with and without the three-way interaction [*X*^2^(1) =.006, *p* = .938]. Moreover, the R^2^_Conditional_ for the two models was the same: .78. Hence, the variance explained by the three-way interaction seems to be very small, providing further evidence for the absence of differences between sweat applications with regard to the linear effect of time.
